# Single Axillary Incision Versus Triple Lateral Chest Wall Incisions in Endoscopic Mastectomy for Gynecomastia: A Single-Center Retrospective Analysis with Propensity Score Matching

**DOI:** 10.1007/s00266-025-04666-6

**Published:** 2025-01-21

**Authors:** Chenhui Xu, Yirui Diao, Ruifu Chen, Meilan Chen, Baoyong Lai

**Affiliations:** 1 Department of Breast Surgery, Xiamen Hospital of Traditional Chinese Medicine, No. 1739 Xianyue Road, Xiamen, 350001 China; 2 Beijing University of Chinese Medicine Third Affiliated Hospital, No. 51 Xiaoguan Street, Andingmen Wai, Chaoyang District, Beijing, 100029 China

**Keywords:** Gynecomastia, Endoscopic mastectomy, Propensity score matching, Visual analog scale, Vancouver scar scale

## Abstract

**Background:**

Endoscopic mastectomy has gradually become an important surgical modality for the treatment of breast diseases, and is the preferred procedure for gynecomastia. However, endoscopic mastectomy presents challenges such as a steep learning curve, prolonged surgical duration, increased hospitalization costs, and high technical difficulty. This study aimed to evaluate the clinical efficacy and patient satisfaction of endoscopic mastectomy using a single axillary incision versus a triple lateral chest wall incision for gynecomastia.

**Methods:**

Patients were stratified into a single-port group and a three-port group based on the surgical approach. Propensity score matching was used for the nearest neighbor matching, adjusting baseline data differences at a 1:1 ratio, with a caliper value set at 0.2 to ensure comparability between the two groups. Clinical efficacy and patient satisfaction were compared after propensity score matching.

**Results:**

A total of 36 pairs of patients were successfully matched after propensity score matching, with no differences in baseline characteristics (*P* > 0.05). Notably, the three-port group experienced longer surgical durations compared to the single-port group, alongside higher hospitalization costs (*P* < 0.05). There were no differences in surgical bleeding volume, postoperative drainage volume, extubation time, postoperative hospitalization time , surgical complications, visual analog scale pain scores, and recurrence rate (*P* > 0.05). After a 6-month follow-up, the vancouver scar scale assessment showed no differences in scar color, thickness, vascularity, softness, and total score (*P* > 0.05). Based on the BODY-Q questionnaire chest module scores, the single-port group showed better overall satisfaction in appearance (*P* = 0.038), especially in the smoothness of the chest wall, with significantly higher scores than the three-port group (*P* = 0.001). No differences were found in nipple symmetry, nipple sensation, and skin redundancy (*P* > 0.05).

**Conclusion:**

The single axillary incision endoscopic mastectomy demonstrated advantages in shorter surgical duration and lower hospitalization costs, while providing a smoother chest wall appearance, thereby enhancing overall patient satisfaction. Consequently, this surgical approach may arise as one of the preferred procedures for gynecomastia.

**Level of Evidence II:**

This journal requires that authors assign a level of evidence to each article. For a full description of these Evidence-Based Medicine ratings, please refer to the Table of Contents or the online Instructions to Authors www.springer.com/00266.

**Supplementary Information:**

The online version contains supplementary material available at 10.1007/s00266-025-04666-6.

## Introduction

Gynecomastia, also known as male breast hypertrophy or male breast development abnormality, is a clinical condition characterized by abnormal proliferation of glandular tissue and excessive growth of connective tissue. Its pathogenesis primarily involves an imbalance between testosterone and estrogen levels, with reported prevalence rates approximately 32–65% [1]. This condition can lead to social anxiety and, sometimes pain or tenderness, impacting patients' life quality [2]. For cases of gynecomastia that do not resolve spontaneously, both medical treatment and surgical intervention are avilable approach [3]. Additionally, gynecomastia may precipitate psychological issues such as depression, anxiety, and inferiority, along with physical changes like pectus carinatum (pigeon chest) and rounded shoulders [4]. Surgical intervention is considered the most effective method for correcting breast contour, improving physical function, and significantly enhancing mental health [5, 6].

Endoscopic mastectomy has gained widespread acceptance due to its minimal invasiveness, faster recovery time, fewer complications, smaller incision scars, and superior cosmetic outcomes [7]. With the continuous advancement of laparoscopic technology and related instruments, various surgical approaches have emerged, providing more options for clinical treatment. This study retrospectively analyzes the clinical efficacy and patient satisfaction of single axillary incision versus triple lateral chest wall incision endoscopic mastectomy in treating gynecomastia, aiming to provide scientific evidence and practical guidance for surgical decision-making.

## Materials and Methods

### General Information

This retrospective analysis included 105 male patients diagnosed with gynecomastia who underwent endoscopic mastectomy at the Department of Breast Surgery, Xiamen Hospital, Beijing University of Chinese Medicine, from January 2014 to December 2023. The inclusion criteria were: meeting the diagnostic criteria for gynecomastia; absence of surgical contraindications; ineffective pharmacotherapy; significant psychological impact and a strong desire for surgical reshaping; and classification as Simon grade IIb or III [8]. Exclusion criteria included: severe organ dysfunction or coagulopathy precluding surgery; severe psychiatric disorders; pseudo-gynecomastia, breast cancer, testicular tumors, and primary hypogonadism.

Patients were divided into two groups based on the surgical approach: the single-port group (n=42) and the three-port group (n=63). All surgeries were performed by the surgical team led by Dr. Xu, with the surgical approach determined jointly by the physicians and patients. The surgeries for both groups were performed in the same time period, according to their respective group assignments.

### Surgical Methods

#### Grouping of Patients

Patients in this study were divided into two groups based on the surgical technique employed. One group underwent endoscopic mastectomy without lipolysis through a single axillary incision (referred to as the single-port group), while the other group underwent endoscopic mastectomy combined with lipolysis and liposuction through three incisions on the lateral chest wall (referred to as the three-port group).

#### Preoperative Preparation

Standard preoperative marking was performed using the "double-ring method" to delineate the boundaries between the glandular and peripheral areas.

1. The inner ring: With the patient in a supine position, the boundaries of the glandular tissue were accurately located using ultrasound and the central glandular area was marked.
2. The outer ring: The patient was then positioned standing to mark the area of chest prominence, including the surrounding adipose tissue, to determine the peripheral area to be excised during surgery.

#### Surgical Methods

##### Single-Port Group Surgical Steps

**Fig. 1:**
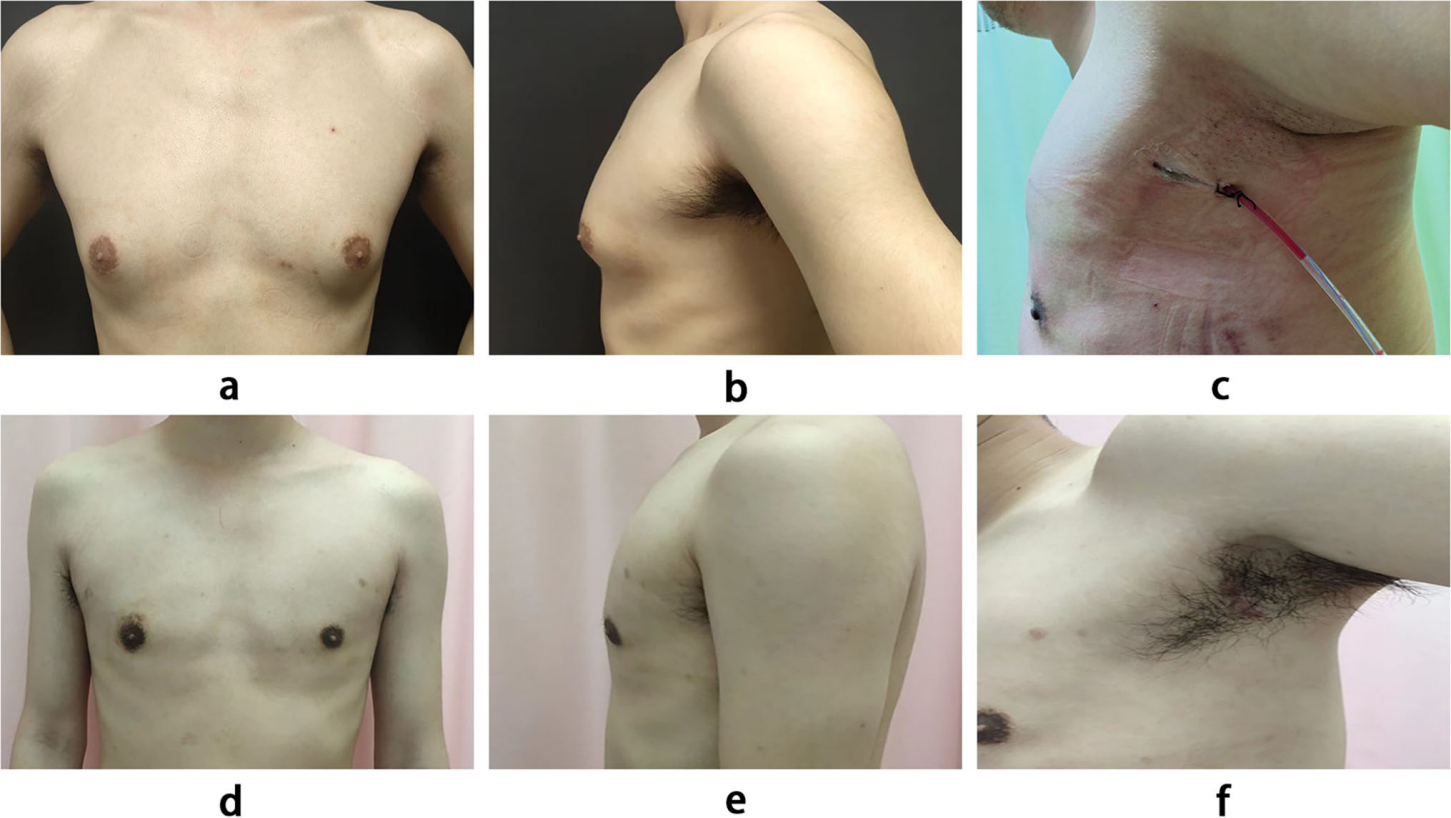
Photographs of endoscopic mastectomy in the single-port group: **a** Preoperative anterior view, **b** Preoperative lateral view (left side), **c** Postoperative incision at two days (left side), **d** Postoperative anterior view, **e** Postoperative lateral view (left side), and **f** Postoperative incision at half year (left side)

1. *Anesthesia and Positioning*: Under general anesthesia, the patient is placed in a supine position with the chest elevated. The upper limbs are abducted and positioned beside the head. Standard disinfection and draping are performed.
2. *Incision and Cavity Creation*: An incision approximately 2.5 cm in length is made at the first skin crease in the axilla, ensuring the anterior edge does not extend beyond the anterior axillary line. After incising the skin and subcutaneous tissue, the space behind the breast is dissected towards the lateral edge of the pectoralis major muscle using an electrocautery. A skin incision retractor (Jiangsu Suyun, B-60/70-150) is placed, and an outer layer of a size 6 sterile glove is used to create a sealed cavity. Through the finger ends of the glove, three 5mm trocars are inserted, designated for the endoscope, electrocautery hook, and operating forceps. CO_2_ is insufflated to create an air space between the skin and the pectoral muscle, maintaining a pressure of approximately 8 mmHg with a flow rate of about 20 L/min.
3. *Gland Dissection*: Under direct endoscopic vision, the retroglandular space of the breast is fully dissected along the surface of the pectoralis major fascia using an electrocautery hook. Subsequently, the fatty connective tissue between the skin and the mammary gland is dissected in the upper outer, upper inner, and lower outer quadrants. Endoscopic tissue scissors are used to sharply transect the glandular tissue and mammary ducts behind the nipple, while preserving a 0.5 cm thickness of glandular tissue behind the nipple. Finally, the fatty connective tissue in the lower inner quadrant is dissected until the mammary gland is completely freed.
4. *Gland Removal and Irrigation Hemostasis*: The excised mammary tissue is removed through the axillary incision. The wound cavity is irrigated with saline under endoscopic vision, meticulous hemostasis is performed, and a negative pressure drainage tube is placed (Fig. 1).

##### Three-Port Group Surgical Steps

**Fig. 2:**
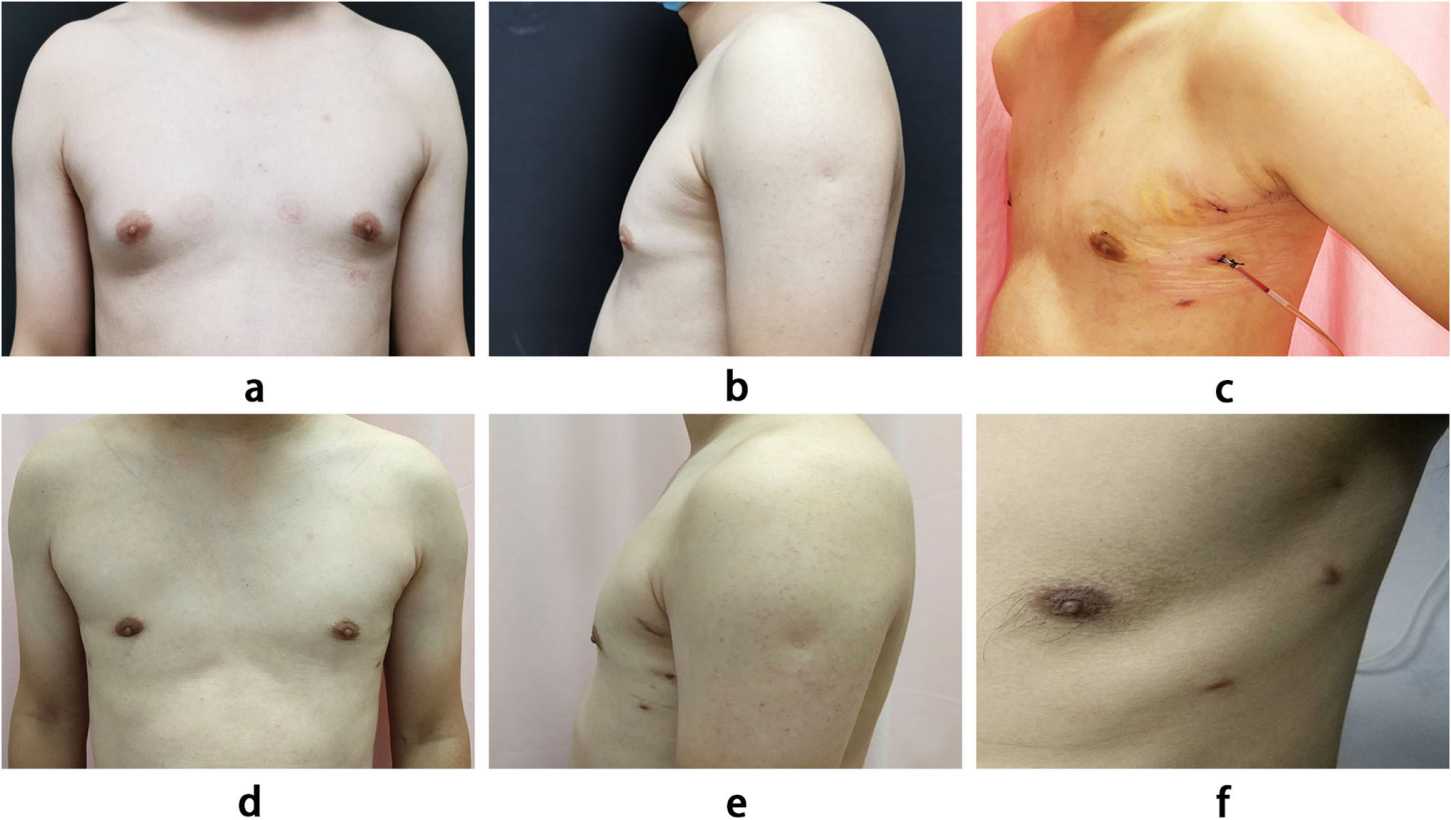
Photographs of endoscopic mastectomy in the three-port group: **a** Preoperative anterior view, **b** Preoperative lateral view (left side), **c** Postoperative incision at two days (left side), **d** Postoperative anterior view, **e** Postoperative lateral view (left side), and **f** Postoperative incision at half year (left side)

1. *Anesthesia and Positioning*: Under general anesthesia, the patient is placed in a supine position with the chest elevated. The upper limbs are abducted and positioned beside the head. Standard disinfection and draping are performed.
2. *Preparation and Injection of Lipolytic Solution*: A lipolytic solution is prepared by mixing 250 ml of normal saline, 250 ml of sterile water for injection, 20 ml of 0.2% lidocaine, and 1 ml of 0.1% epinephrine. The solution is injected uniformly into the subcutaneous fat layer and the retroglandular space through a puncture at the edge of the areola, with 300 mL and 150 mL injected respectively. The breast is manually massaged for approximately 20 minutes to ensure the solution fully acts on the tissues.
3. *Liposuction and Cavity Creation*: A No. 6 curette is connected to a negative pressure suction device to thoroughly aspirate the fat tissue from the subcutaneous layer and retromammary space. During aspiration, the suction device is oriented towards the glandular tissue, avoiding the chest wall skin and muscles, maintaining uniform suction until the aspirate turns light red. A longitudinal incision of about 10 mm is made at the level of the nipple on the mid-axillary line to serve as the endoscopic observation port, and two incisions of about 5 mm are made at the upper and lower edges of the marked outer ring on the mid-axillary line to serve as working ports. Trocars with diameters of 10 mm, 5 mm, and 5 mm were inserted through these incisions. CO2 is insufflated to create an air space between the skin and the pectoral muscle, maintaining a pressure of approximately 8 mmHg with a flow rate of about 20 L/min.
4. *Gland Dissection*: Under direct endoscopic vision, the minimal fibrous connective tissue of the retromammary space is dissected to the edge of the gland using an electrocautery hook. Subsequently, the Cooper's ligaments between the skin and the gland are transected towards the nipple. When dissecting posterior to the nipple, the glandular tissue and mammary ducts behind the nipple are sharply transected using endoscopic tissue scissors, taking care to preserve the subareolar vascular network.
5. *Gland Removal and Irrigation Hemostasis*: The mammary gland tissue is cut into strips and completely removed through the endoscopic observation port. The wound cavity is irrigated with saline under endoscopic vision to ensure no residual tissue fragments or blood. Meticulous hemostasis is performed, and a negative pressure drainage tube is placed (Fig. 2).

#### Postoperative Care

Postoperatively, closely monitor the blood supply to the nipple, areola, and skin flaps, and record the color and amount of drainage fluid in detail. The drainage tube is promptly removed when the volume of drainage fluid is reduced to below 20 ml. A moderate pressure bandage with a chest wrap is applied to the breast surface. No prophylactic antibiotics were administered to patients postoperatively. Patients who do not experience complications and meet the discharge criteria are scheduled for discharge.

### Efficacy and Evaluation Indicators

The surgical outcomes are evaluated, including unilateral surgical duration, bleeding volume, drainage volume, extubation time, postoperative hospitalization time, and hospitalization costs. Additionally, the occurrence of complications such as subcutaneous effusion, subcutaneous hematoma, ecchymosis, emphysema, nipple-areolar complex necrosis, and postoperative infection is monitored.

In accordance with postoperative care standards, we routinely conduct assessments of surgical pain. The clinical pain level of patients is assessed using the Visual Analogue Scale (VAS) on postoperative days 1, 2, and 3. The VAS ranges from 0 to 10, with higher scores indicating greater pain intensity.

### Follow-Up

At the six-month postoperative mark, we conducted follow-ups with all surgical patients. Treatment efficacy is evaluated by re-examination of color Doppler ultrasound to assess whether there is a recurrence of mammary gland tissue. The incision healing is comprehensively evaluated using the Vancouver Scar Scale (VSS) [9], which assesses scar color, thickness, vascularity, and pliability, with a total score of 15 points. A higher score indicates poorer scar healing. Based on the chest module scoring standards of the BODY-Q questionnaire [10], and in conjunction with preoperative and postoperative photographs. A multi-dimensional evaluation of breast appearance is conducted using the observer-reported method, involving the chest contour evenness, nipple symmetry, nipple sensation, and skin redundancy, with each item scored from 1 to 4 points, and a total score of 16 points, where a higher score indicates greater patient satisfaction with the appearance.

### Statistical Methods

Statistical analysis is performed using SPSS 25.0 software. Normally distributed measurement data are presented as mean ± standard deviation (x ± s), and intergroup comparisons are conducted using the independent samples t-test. Non-normally distributed data are presented as medians with interquartile ranges [M (P25-P75)], and intergroup comparisons are performed using nonparametric rank-sum tests. Count data are presented as frequencies and percentages (%), and intergroup comparisons are conducted using chi-square test or Fisher's exact test. All P-values are two-sided, and statistical significance is set at *P* < 0.05. To mitigate potential baseline data differences, Propensity Score Matching (PSM) is employed with a 1:1 ratio and a caliper width of 0.2 to enhance comparability between the two groups.

## Results

### Baseline Characteristics of Patients in Both Groups (See Table 1)

**Table 1:** Comparison of baseline characteristics in both patient groups

Variable	Before PSM		Statistical value	*P* value	After PSM		Statistical value	*P* value
	single-port group (n=40)	three-port group (n=63)			single-port group (n=36)	three-port group (n=36)		
Age (years)	27 (21.8–34)	29 (25–34)	1.601	0.109^b^	26.5 (21.3–33.8)	30.5 (26–34)	1.905	0.057^b^
BMI (kg/m^2^)	25.11±3.74	26.67±3.51	2.172	0.032^a^	25.34±3.60	26.06±3.73	0.848	0.410^a^
Smoking, n (%)	12 (28.6%)	10 (15.9%)	2.453	0.117^c^	11 (33.6%)	7 (19.4%)	1.185	0.276^c^
Pituitary tumor, n (%)	4 (9.5%)	2 (3.2%)	0.891	0.345^c^	3 (8.3%)	2 (5.6%)		1.000^d^
Diabetes, n (%)	5 (11.9%)	4 (6.3%)	0.410	0.522^c^	5 (13.9%)	2 (5.6%)		0.429^d^
Disease duration (years)	5 (3–10)	5 (2–10)	0.291	0.771^b^	5 (3–10)	5.5 (2.3–10)	0.263	0.793^b^
Side			1.352	0.245^c^				0.151^d^
Unilateral, n (%)	5 (11.9%)	13 (20.6%)			2 (5.6%)	7 (19.4%)		
Bilateral, n (%)	37 (88.1%)	50 (79.4%)			34 (94.4%)	29 (80.6%)		
Simon type			1.191	0.275^c^			3.130	0.077^c^
IIb, n (%)	17 (40.5%)	19 (30.2%)			15 (41.7%)	8 (22.2%)		
III, n (%)	25 (59.5%)	44 (69.8%)			21 (58.3%)	28 (77.8%)		
Ultrasound								
Diameter (mm)	54.98±13.98	57.62±13.39	0.974	0.333^a^	55.58±14.01	58.36±13.06	0.536	0.387^a^
Thickness (mm)	10 (9–12)	11 (10–14)	1.613	0.107^b^	10 (9–12)	11 (9–14)	0.918	0.358^b^
E2 (pmol/l)	144.40±44.34	125.13±40.52	2.299	0.024^a^	137.82±39.61	134.63±39.89	0.340	0.735^a^
T (nmol/l)	15.77±4.36	13.93±4.44	2.098	0.038^a^	15.33±3.85	15.14±4.58	0.331	0.847^a^
PAL (mIU/L)	207.9 (165.0–274.3)	186.3 (136.3–232.4)	2.087	0.037^b^	199.6 (163.4–254.3)	182.2 (136.2–232.0)	1.633	0.102^b^

^a^Independent samples t-test, ^b^Nonparametric rank sum test, ^c^Chi-squared test, ^d^Fisher's exact probability method

*PSM* Propensity score matching, *BMI* Body mass index, *E*_*2*_ Estrogen, *T* Testosterone, *PAL* Prolactin.

This retrospective analysis included 105 patients. And patients were divided into two groups based on the surgical approach: the single-port group (n=42) and the three-port group (n=63). Prior to Propensity Score Matching (PSM), there were statistically significant differences in BMI, T, and PAL levels between the two groups (*P*<0.05). After PSM, 36 pairs of patients were successfully matched, and comparisons of all baseline characteristics showed no statistically significant differences (*P*>0.05).

### Efficacy and Evaluation Indicators in Both Groups (See Table 2)

**Table 2:** Comparison of clinical indicators in both patient groups

Factor		Single-port group (n=36)	Three-port group (n=36)	Statistical value	*P* value
Unilateral surgical duration (min)		111.0 (97.5–124.25)	120.25 (104.75–132.375)	2.118	0.034^b^
Unilateral bleeding volume (ml)		15 (10–25)	17.5 (10.0–25.0)	0.246	0.806^b^
Unilateral drainage volume (ml)		78.75 (66.875–107.0)	91 (77–124)	1.639	0.101^b^
Extubation time (days)		5 (4.25–6)	6 (5–6)	1.190	0.234^b^
Postoperative hospitalization time (days)		5 (3–6)	5 (3.25–6)	0.034	0.973^b^
Hospitalization cost (thousand RMB)		14.2±2.1	15.5±2.7	2.281	0.026^a^
Subcutaneous effusion, n (%)		2 (5.6%)	7 (19.4%)		0.151^d^
Subcutaneous hematoma, n (%)		0	0		
Skin ecchymosis, n (%)		4 (11.1%)	1 (2.8%)		0.357^d^
Subcutaneous emphysema, n (%)		0	1 (2.8%)		1.000^d^
Nipple-areola complex necrosis, n (%)		0	0		
Postoperative infection, n (%)		0	0		
Visual analogue scale, postoperative day	1	4 (4–5)	4 (4–4)	1.837	0.066^b^
	3	3 (2–3)	3 (2–3)	1.424	0.154^b^
	5	2 (2–2)	2 (1–2)	1.183	0.237^b^
Postoperative recurrence, n (%)		0	1 (2.8%)		1.000^d^

^a^Independent samples t-test, ^b^Nonparametric rank sum test, ^c^Chi-squared test, ^d^Fisher's exact probability method

#### Surgical Outcomes

All surgeries in both groups were successfully completed, with no conversions to open surgery. After PSM, there were no statistically significant differences in surgical bleeding volume, postoperative drainage volume, extubation time, and postoperative hospitalization time between the two groups (*P*>0.05). However, the Single-Port Group demonstrated a significant advantage in terms of surgical duration and hospitalization costs: the average surgical duration was shorter compared to the Three-Port Group (111 minutes vs. 120.25 minutes, *P* = 0.034), and the hospitalization costs were also lower (CNY 14.2 ± 2.1 thousand vs. CNY 15.5± 2.7 thousand, *P* = 0.026), with both differences reaching statistical significance (*P* < 0.05).

#### Complications

After PSM, there were no statistically significant differences in the incidence of common complications between the two groups (P>0.05). Nevertheless, subcutaneous effusion occurred in 7 cases in the Three-Port Group and only 2 cases in the Single-Port Group; ecchymosis was observed in 1 case in the Three-Port Group compared to 4 cases in the Single-Port Group; and subcutaneous emphysema occurred in 1 case in the Three-Port Group, with no such complications observed in the Single-Port Group. No cases of hematoma, nipple-areola complex necrosis, or infection were observed in either group.

#### Postoperative Pain Scores

Using the Visual Analogue Scale (VAS), pain scores were assessed on postoperative days 1, 3, and 5. The results showed no statistically significant differences in postoperative pain scores between the two groups (P>0.05), and the pain levels decreased gradually over time.

#### Follow-up

During the 6-month follow-up, one case of recurrence was identified in the Three-Port Group upon re-examination with color Doppler ultrasound, whereas no recurrences were observed in the Single-Port Group. However, the difference between the two groups was not statistically significant (*P* > 0.05).

### Surgical Scar and Breast Appearance Satisfaction in Both Groups (See Table 3)

**Table 3:** Comparison of surgical scar and breast appearance satisfaction in both patient groups

Factor		Single-port group (n=36)	Three-port group (n=36)	Z value	*P* value
Vancouver scar scale	Total score (0–15 points)	6 (5–7.75)	6 (4.25–6.75)	0.986	0.324^a^
	Color (0–3 points)	1 (1–2)	1 (1–2)	0.471	0.638^a^
	thickness (0–4 points)	2 (1–2)	1 (1–2)	1.160	0.246^a^
	Vascularity (0–3 points)	1 (1–1.75)	1 (1–2)	0.772	0.440^a^
	Pliability (0–5 points)	2 (1–2)	2 (1–2)	0.868	0.385^a^
Appearance satisfaction scores	Total score (4–16 points)	13 (12–14)	12.5 (12–13.75)	2.078	0.038^a^
	Chest contour evenness (1–4 points)	3 (3–4)	3 (3–3)	3.244	0.001^a^
	Nipple symmetry (1–4 points)	3 (3–4)	3 (3–4)	0.323	0.747^a^
	Nipple sensation (1–4 points)	3 (3–3)	3 (3–3)	0.951	0.341^a^
	Skin redundancy (1–4 points)	4 (3–4)	3 (3–4)	0.759	0.448^a^

^a^Nonparametric rank sum test

#### Surgical Scar Score

During the 6-month follow-up, the Vancouver Scar Scale (VSS) was used to meticulously assess the degree of surgical scars. There were no statistically significant differences in the color, thickness, vascular distribution, softness, and total score of the surgical incision scars between the two groups (*P*>0.05).

#### Breast Appearance Satisfaction Scores

Breast appearance satisfaction was assessed using the chest module of the BODY-Q questionnaire, combined with preoperative and postoperative photograph comparisons, and scored through an observer-reported method. The overall breast appearance satisfaction score was significantly higher in the Single-Port group compared to the Three-Port group (*P*=0.038), especially in the smoothness of the chest wall, where the Single-Port Group was significantly better than the Three-Port Group (*P*=0.001). No statistically significant differences were observed between the two groups in nipple symmetry, nipple sensation, or skin redundancy (*P*>0.05).

## Discussion

In 1538, Paulus Aeginea [8] first reported the employment of surgical intervention for gynecomastia. Since then, various techniques have been developed, including liposuction, open surgery, and endoscopic approaches, all of which still have some unsatisfactory aspects [12]. Traditional open surgical techniques, with or without liposuction, have proven reliable for treating gynecomastia. Common incision sites include periareolar, transareolar, and inframammary fold locations [13]. While these incisions effectively remove hyperplastic glandular tissue and/or excess skin, they can leave conspicuous scars around the areola [14, 15], negatively impacting patients' physical appearance and potentially their psychological well-being [16]. Traditional open surgical methods are associated with several complications, such as breast contour irregularities, hematoma, seroma, numbness, necrosis, breast asymmetry, and dissatisfaction with cosmetic outcomes [17]. Furthermore, periareolar and transareolar incisions carry a significant risk of damaging the subdermal neurovascular plexus of the nipple-areola complex (NAC), leading to hypoesthesia or NAC necrosis [18]. Goh et al. introduced the microdebrider excision combined with liposuction technique in 2010 [19], and they found that the most common complication of this approach was residual breast disc (11.5%) [20]. Under-resection is a frequent issue with liposuction-only procedures [21]. The lower satisfaction rates observed with power-assisted liposuction may be attributed to a higher percentage of residual breast disc (19.2%), which results in a higher reoperation rate [22].

Ohyama et al. [23] successfully performed the first endoscopic-assisted mastectomy, marking a novel avenue in the management of gynecomastia. Jarrar and colleagues [24] explored lipolysis-assisted liposuction for gynecomastia, yet standalone liposuction treatments were associated with high reoperation rates due to residual glandular tissue [21]. Our previous report on endoscopic mastectomy combined with liposuction [7] offered patients superior therapeutic effects, characterized by minimal trauma, rapid recovery, fewer complications, inconspicuous scars, and excellent cosmetic results. With the further development of minimally invasive techniques such as endoscopy, various surgical approaches have emerged. Varlet et al. [12] and Yang et al. [25] respectively, utilized a combination of one 10-mm and two 5-mm incisions, as well as a single axillary incision for endoscopic mastectomy combined with liposuction for gynecomastia, both achieving satisfactory cosmetic results. Nevertheless, comparative studies evaluating different surgical approaches for endoscopic mastectomy remain scarce. This study aims to compare the clinical efficacy of single axillary incision versus three-port lateral chest wall incision endoscopic mastectomy for gynecomastia, providing a reference for the selection of surgical methods.

Propensity score matching (PSM) is an effective tool in mitigating baseline data bias, allowing for more reasonable comparisons between groups [26]. In this study, PSM was used to balance the differences in baseline data such as BMI, T, and PAL between the three-port group and the single-port group. A 1:1 nearest neighbor matching yielded 36 matched pairs, ensuring no statistically significant differences in baseline characteristics. Following propensity score matching, our study's findings revealed the following:

1. Both surgical approaches were successfully executed without any conversions to open surgery. There were no statistically significant differences in surgical bleeding volume, postoperative drainage volume, extubation time, and postoperative hospitalization time between the two groups. Varlet et al. [27] performed endoscopic mastectomy combined with liposuction for 24 adolescent patients with grade 2B-3 gynecomastia, with an average surgical duration of 160 minutes, while Tukenmez et al. reported an average surgical duration of 120 minutes for 30 patients undergoing single axillary incision endoscopic mastectomy. They found that an axillary incision permits direct extraction of mammary gland without the need to fragment it, thereby shortening the surgical duration [28]. Lipolysis and liposuction are the primary method of cavity construction in endoscopic mastectomy [29], but they require a certain amount of time. This study compared the surgical duration of the single axillary incision endoscopic mastectomy without lipolysis (single-port group) to the triple-incision endoscopic mastectomy combined with lipolysis and liposuction on the lateral chest wall (three-port group). The results showed that the single-port group had a shorter surgical duration than the three-port group (111 minutes vs. 120.25 minutes, *P* = 0.034), consistent with the aforementioned studies. In the single-port group was unnecessary to cut the breast tissue into strips for removal during surgery, and it could be directly and completely removed through the axillary incision. Moreover, the single-port group employed a lipolysis-free method to directly separate the subcutaneous adipose tissue and the retromammary space, avoided lipolysis and liposuction steps, which saved a significant amount of time compared to the three-port group.
  Longer surgical duration increased anesthesia requirements, and additional liposuction procedures, both contributing to higher hospitalization costs. Our results demonstrated that the single-port group had significantly lower hospitalization expenses than the three-port group (CNY 14.2 ± 2.1 thousand vs. CNY 15.5± 2.7 thousand, *P* = 0.026).
2. Common complications following endoscopic mastectomy include subcutaneous effusion, hematoma, ecchymosis, emphysema, nipple-areola complex necrosis, and infection [30]. Holzmer et al. [31] reported an overall postoperative complication rate of 13.1%, including infection, nipple sensation changes, skin redundancy, nipple-areola necrosis, hematoma, and subcutaneous effusion. Among these, hematoma formation is the most common early complication, with an incidence rate of 2%-15%, which may increase the risk of infection [32]. In our study, there were no statistically significant differences in the incidence of common postoperative complications between the groups. This can be attributed to the magnifying effect of endoscopic surgery, meticulous operation, precise hemostasis, and the application of postoperative compression dressings, which resulted in only mild ecchymosis in a few patients and no hematoma cases. As the main method for establishing access in endoscopic mastectomy [29], liposuction provides clear anatomical structures, requiring only the division of Cooper's ligaments, without the need for separation in the fatty interspaces. However, the injection of lipolytic fluid may increase the risk of subcutaneous effusion. In our study, seven cases of subcutaneous effusion (19.4%) occurred in the three-port group, whereas only two cases (5.6%) were noted in the single-port group. Despite these observations, the difference in the incidence of subcutaneous effusion and extubation time was not statistically significant. Innocenti et al. [33] reported a 1.92% rate of nipple-areola complex necrosis related to liposuction techniques. Other study [34] suggest that an axillary incision approach for endoscopic mastectomy better preserves the blood supply to the nipple-areola complex. Therefore, we consistently preserved a 5mm layer of breast tissue behind the nipple and used sharp dissection with scissors to avoid the risk of thermal injury to the nipple from electrocautery. Consequently, neither group experienced nipple-areola complex necrosis postoperatively.
3. In terms of postoperative pain management, this study utilized the Visual Analog Scale (VAS) to assess pain levels on the first, third, and fifth days following surgery. There were no significant differences in pain scores between the two groups. The lidocaine present in the lipolytic solution used in the three-port group provided local anesthetic effects, which likely contributed to slightly lower pain scores compared to the single-port group. As wound healing progressed and recovery ensued, pain scores gradually declined in both groups.
4. Both surgical approaches showed equivalent effectiveness, with no statistically significant differences in surgical efficacy. At the six-month follow-up with color Doppler ultrasound, only one recurrence was found in the three-port group. The patient was a fitness enthusiast, and the recurrence may be related to the postoperative intake of testosterone. Testosterone is an anabolic steroid hormone, and literature indicates that up to 50% of steroid users develop gynecomastia [35, 36]. Babigian et al. [37] noted that 15% of patients experienced recurrence with continued use of anabolic steroids.
5. Psychologically, gynecomastia can elicit anxiety, low self-esteem, depression, and social phobia, significantly impacting patients' mental and physical health [29, 38, 39]. These patients often have high expectations for scar concealment and cosmetic satisfaction, as a hidden scar and an ideal appearance can significantly alleviate psychological burdens and boost self-confidence [38]. Common reasons for patient dissatisfaction with surgery include residual breast tissue, chest wall skin depression, skin redundancy and folds, nipple-areola complex asymmetry, abnormal nipple-areola complex sensation, and postoperative scar formation and contracture [40]. Luo Chengyu [41] proposed the "5S" goals for endoscopic mastectomy: thorough *sweeping* of breast tissue, *scar* concealment, bilateral *symmetry*, normal male chest *shape*, and *smooth* skin appearance. Therefore, an ideal surgery should completely remove the mammary tissue, leave concealed incision scars, achieve bilateral symmetry, present a normal male chest shape, and result in smooth skin postoperatively [42]. This study assessed the degree of surgical incision scars at six months postoperatively using the Vancouver Scar Scale (VSS), with no significant differences between the two groups. Although the single-port group had a longer surgical incision (2.5 cm) compared to the maximum diameter of incisions in the three-port group (1 cm), the fewer number of scars and their location within axillary skin folds, coupled with darker pigmentation and axillary hair coverage, meant that the single-port group was not inferior to the three-port group in terms of scar satisfaction.

Currently, there is a lack of effective tools to assess the aesthetic outcomes following treatment for gynecomastia. Klassen et al. [43] utilized the BODY-Q questionnaire to assess clinically relevant postoperative outcomes, which comprises 26 separate functional scales encompassing appearance satisfaction, health-related quality of life (HRQL), and healthcare experiences. The chest module of BODY-Q is specifically designed for patients undergoing chest contouring procedures, addressing aspects such as the chest, nipples, body image, social functioning, appearance concerns, and scar areas, with scores ranging from 0 to 100. In this study, we adapted the chest module of the BODY-Q questionnaire, combined with preoperative and postoperative photograph comparisons, to assess breast appearance satisfaction using an observer-reported method. The three-port group required liposuction to create a cavity, resulting in subcutaneous tissue in the surgical area being lower than surrounding untreated areas, potentially leading to chest wall irregularity or even "volcanic crater "-like changes [25]. Conversely, the single-port group employed a non-lipolysis approach to create the cavity, separating the skin flap along the breast tissue while preserving subcutaneous fat tissue as much as possible, ensuring a smoother transition of subcutaneous tissue and a superior chest wall flatness compared to the three-port group. Consequently, the overall appearance satisfaction score was significantly higher in the single-port group. However, there were no statistically significant differences between the two groups in terms of nipple symmetry, nipple sensation, and skin redundancy. The incidence of nipple sensation reduction in endoscopic mastectomy ranges from 3 to 19.2% [6]. The nerves of the nipple-areola complex originate from the lateral and anterior cutaneous branches of the fourth intercostal nerve, with the superficial branches mainly distributed in the fascial fatty layer overlying the glandular surface. Using scissor technique to dissect the tissue behind the nipple, preserving about 5 millimeters of tissue, not only prevents postoperative depression and deviation of the nipple-areola area but also protects the subcutaneous vascular network and nerves, minimizing the reduction of nipple sensation. Some scholars [31] have suggested that patients with Simon grades IIB-III may experience skin redundancy, necessitating skin excision. However, Hammond et al. [44] argue that the skin elasticity in younger patients is strong, allowing for consideration of further skin treatment after stabilization of postoperative results, which could reduce skin excision and unnecessary scarring. Varlet et al. [12] and Fan et al. [45] used endoscopic mastectomy to treat grade IIb and III gynecomastia, and this study adopted a similar method, achieving a high level of appearance satisfaction. Ultimately, compared with skin redundancy, patients may be more concerned about the impact of scars on aesthetics.

This study also has several limitations. Firstly, the follow-up period is not long enough, focusing primarily on short-term outcomes, particularly complications and patient satisfaction. Long-term follow-up data, including long-term aesthetic effects and quality of life reports, will provide a more comprehensive evaluation. Secondly, there is currently a lack of appropriate tools to assess the aesthetic effects after surgery. To address this gap, we referenced the chest module of the BODY-Q questionnaire, which is validated for measuring patient-reported outcomes, and utilized observer-reported assessments to strengthen the the validity and reliability of our findings. Thirdly, as a single-center retrospective study, while propensity score matching reduced the impact of selection bias and confounding factors, it may have resulted in the loss of some observations, reducing the sample size and representativeness. Therefore, subsequent studies should employ large-sample, multi-center, prospective, randomized trials to further validate the conclusions drawn here.

## Conclusion

Single-axillary incision endoscopic mastectomy for gynecomastia represents a safe and effective approach. It offers numerous advantages, including minimal trauma, less pain, rapid recovery, fewer complications, inconspicuous scars, and excellent cosmetic results. Compared to the three-incision lateral chest wall endoscopic mastectomy, the single-axillary incision approach avoids lipolysis and liposuction, allows for the intact removal of surgical specimens, shortens the surgical duration, decreases hospitalization costs, and enhances the aesthetic outcome with a smoother chest shape and higher satisfaction with appearance.

## Supplementary Information

Below is the link to the electronic supplementary material.

Supplementary file1 (MP4 440126 KB)

Supplementary file2 (MP4 475209 KB)
